# 2,2′-Dimethyl-5,5′-dipropan-2-yl-4,4′-(phenyl­methyl­ene)diphenol

**DOI:** 10.1107/S1600536810033441

**Published:** 2010-08-25

**Authors:** Ahmad Oubair, Rachid Fihi, Lhou Majidi, Mohamed Azrour, Jean-Claude Daran

**Affiliations:** a Laboratoire des Substances Naturelles & Synthèse et Dynamique Moléculaire, Faculté des Sciences et Techniques, BP 509, Errachidia, Morocco; b Laboratoire de Chimie Physique des Matériaux, Faculté des Sciences et Techniques, BP 509, Errachidia, Morocco; c Laboratoire de Chimie de Coordination, UPR-CNRS 8241, 205 route de Narbonne, 31077 Toulouse Cedex, France

## Abstract

In the title mol­ecule, C_27_H_32_O_2_, the aromatic rings are in a propeller configuration. In the crystal, mol­ecules are linked through O—H⋯O hydrogen bonds forming a two-dimensional network which develops parallel to (010). Futhermore, weak C—H⋯π inter­actions involving the two substituted rings build up a three-dimensional network.

## Related literature

*R*-(−)-Carvone, *p*-mentha-6,8-dien-2-on, is the major constituent of spearmint essential oil of *Menthe spicata* (Gershenzon *et al.*, 1989 ▶) and is an important chiron for the synthesis of complex natural products (Wang *et al.*, 2001 ▶) and anti­viral agents. We have reported an efficient method which affords direct access to *p*-cymene derivatives from *R*-(−)-carvone, see: Majidi & Fihi (2004 ▶). For our inter­est in the development of strategies for the synthesis of natural product derivatives, see: Majidi *et al.*, 2005 ▶). For related structures, see; Guo *et al.* (2005 ▶); Sarma & Baruah (2004 ▶, 2005 ▶); Veldman *et al.* (1996 ▶); Yang *et al.* (2005 ▶).
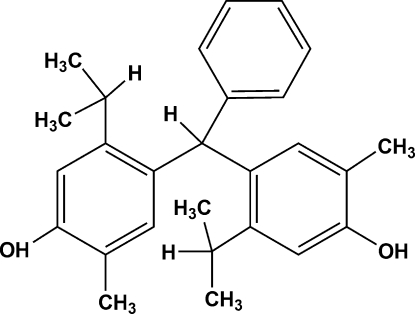


## Experimental

### 

#### Crystal data

C_27_H_32_O_2_
*M* *_r_* = 388.53
Monoclinic, 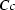

*a* = 11.3775 (7) Å
*b* = 24.6369 (11) Å
*c* = 8.8687 (6) Å
β = 112.913 (8)°
*V* = 2289.8 (2) Å^3^
*Z* = 4
Mo *K*α radiation
μ = 0.07 mm^−1^
*T* = 180 K
0.55 × 0.35 × 0.11 mm

#### Data collection

Oxford Diffraction Xcalibur diffractometer
Absorption correction: multi-scan (*CrysAlis RED*; Oxford Diffraction, 2006 ▶) *T*_min_ = 0.723, *T*_max_ = 1.000
10216 measured reflections
2838 independent reflections
1792 reflections with *I* > 2σ(*I*)
*R*_int_ = 0.048

#### Refinement

*R*[*F* ^2^ > 2σ(*F* ^2^)] = 0.046
*wR*(*F* ^2^) = 0.103
*S* = 0.95
2838 reflections
269 parameters
2 restraints
H-atom parameters constrained
Δρ_max_ = 0.32 e Å^−3^
Δρ_min_ = −0.36 e Å^−3^

### 

Data collection: *CrysAlis CCD* (Oxford Diffraction, 2006 ▶); cell refinement: *CrysAlis RED* (Oxford Diffraction, 2006 ▶); data reduction: *CrysAlis RED*; program(s) used to solve structure: *SIR97* (Altomare *et al.*, 1999 ▶); program(s) used to refine structure: *SHELXL97* (Sheldrick, 2008 ▶); molecular graphics: *ORTEP-3 for Windows* (Farrugia, 1997 ▶); software used to prepare material for publication: *SHELXL97*.

## Supplementary Material

Crystal structure: contains datablocks I, global, azrour7. DOI: 10.1107/S1600536810033441/pv2319sup1.cif

Structure factors: contains datablocks I. DOI: 10.1107/S1600536810033441/pv2319Isup2.hkl

Additional supplementary materials:  crystallographic information; 3D view; checkCIF report

## Figures and Tables

**Table 1:** Hydrogen-bond geometry (Å, °) *Cg*2 and *Cg*3 are the centroids of the C21–C26 and C31–C36 rings, respectively.

*D*—H⋯*A*	*D*—H	H⋯*A*	*D*⋯*A*	*D*—H⋯*A*
O24—H24⋯O34^i^	0.84	2.05	2.871 (3)	164
O34—H34⋯O24^ii^	0.84	2.27	3.051 (3)	154
C23—H23⋯O34^i^	0.95	2.51	3.259 (3)	135
C13—H13⋯*Cg*3^iii^	0.95	2.92	3.658 (4)	135
C15—H15⋯*Cg*2^iv^	0.95	2.86	3.790 (5)	167

Symmetry codes: (i) 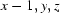; (ii) 
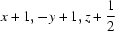
; (iii) 
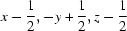
; (iv) 
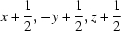
.

**Fig. 1.**
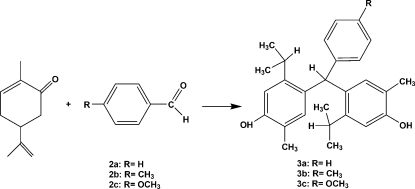
Schematic diagram of the synthetic pathway.

**Fig. 2.**
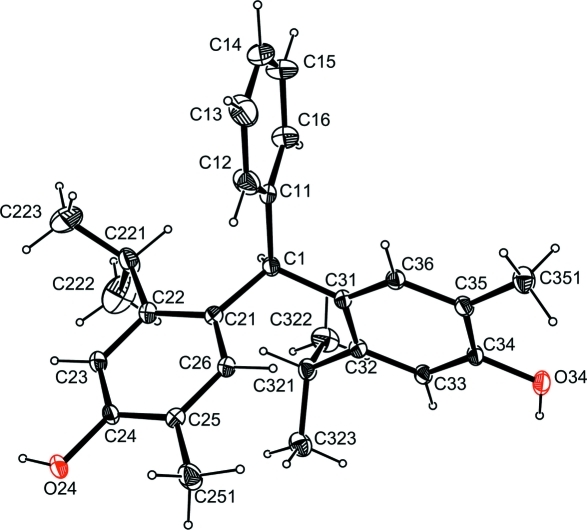
The asymmetric unit of the title molecule with the atom-labelling scheme. Displacement ellipsoids are drawn at the 30% probability level. H atoms are represented as spheres of arbitrary radii.

**Fig. 3.**
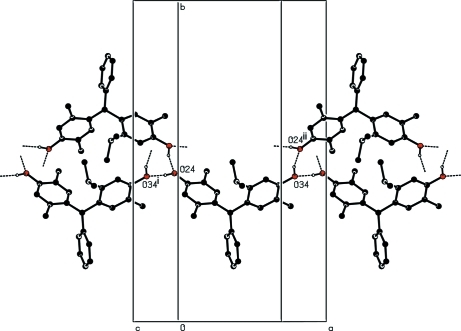
Partial packing view of the title compound, showing the formation of layers parallel to the (0 1 0) plane built from O—H···O hydrogen; H atoms not involved in hydrogen bonding have been omitted for clarity,. [Symmetry codes: (i) x - 1, y, z; (ii) x + 1, -y + 1, z + 1/2]

## supplementary crystallographic information

### Comment

*R*-(-)-Carvone, *p*-mentha-6,8-dien-2-on, is the major constituent of spearmint essential oil of *Menthe spicata* (Gershenzon *et al.*, 1989). This monoterpene ketone is used as a fragrance component and flavouring agent. *R*-(-)-Carvone is also an important chiron for the synthesis of complex natural products (Wang *et al.*, 2001) and antiviral agents. Recently, we reported an efficient method which affords direct access to *p*-cymene derivatives from *R*-(-)-carvone (Majidi & Fihi, 2004). In our continuing interest (Majidi *et al.*, 2005) in the development of strategies for the synthesis of natural products derivatives, we report herein the synthesis of carvacrol derivatives from *R*-(-)-carvone.

The condensation of arylaldehydes (2a-c) to (*R*)-(-)-carvone (1) in acid media at reflux in toluene leads to carvacrol derivatives (3a-c), respectively. Products (3a-c) were obtained by a condensation followed by a rearrangement (Fig. 1). Since the ^1^H and ^13^C NMR studies did not provide unambiguous information on the structure of (3), a single-crystal X-ray study was carried out for the product (3a).

In the molecule of the title compound the phenyl rings are in a propeller configuration with roughly identical dihedral angles between the rings: 88.27 (8)° between the C11-C16 and C21-C26 rings, 85.79 (6) between the C21-C26 and C31-C36 rings and 82.52 (8)° between C31-C36 and C11-C16 rings (Fig. 2). Propeller like arrangement has been observed in several related compounds, e.g., CH(C_6_H_5_)_3_ (Veldman *et al.*, 1996), CH(C_6_H_5_)_2_[C_6_H_2_(OH)_2_CH(C_6_H_5_)_2_](C_6_H_5_CHO) (Guo *et al.*, 2005), CH(C_6_H_5_)(C_6_H_4_OH)_2_,0.17(H_2_O) (Sarma & Baruah, 2005), CH(C_6_H_5_)[C_6_H_2_(CH_3_)_2_OH]_2 _(Sarma & Baruah, 2004), CH(C_6_H_5_)[C_6_H_2_(OH)_2_Cl]_2_,*C*_2_H_6_O,H_2_O and CH(C_6_H_5_)[C_6_H_2_(OCH_3_)_2_Cl]_2_(Yang *et al.*, 2005). The bond distances and angles in the title molecule agree with the corresponding distances and angles reported in the structures quoted above.

In the crystal, the molecules are linked through O—H···O hydrogen bonds involving the donor oxygen atom O24 and the acceptor O34 forming infinite chains. These chains are further connected through weak O—H···O hydrogen bonds involving as donor atom O34 and as acceptor O24 (Table 1) resulting in the formation of a two dimensionnal network developping parallel to the (0 1 0) plane (Fig. 3; Table 1).

Futhermore, weak C—H···π interactions involving the C13 and C15 atoms and the centroids *Cg*2 and *Cg*3 of the C21-C26 and C31-C36 rings, respectively, build up a three dimensionnal network (Table 1).

### Experimental

(*R*)-(-) Carvone (1) is a commercial product. A mixture of carvone (3 g, 2 mmol), corresponding aromatic aldehyde (1.06 g, 10 mmol) in toluene (50 ml) and TsOH.H_2_O (*p*-toluene sulphonic acid hydrate) (0.28 g) was heated under reflux using a Dean-stark trap for 24 h. The reaction mixture was poured into cold water (100 ml), and extracted. The organic phase was washed with water (4 *x* 30 ml), dried (Na_2_SO_4_), and evaporated *in vacuo*. The crude products were purified by column chromatography on silica gel. Eluant: hexane/dichloromethane (60/40). The compound was finally recrystallized from ethanol.

### Refinement

All H atoms attached to C atoms and O atom were fixed geometrically and treated as riding with C—H = 1.0Å (methine), 0.98 Å (methyl) or 0.95 Å (aromatic) and O—H = 0.84 Å with *U*_iso_(H) = 1.2*U*_eq_(C) or *U*_iso_(H)= 1.5*U*_eq_(O, C-methyl).

In the absence of significant anomalous scattering, the absolute structure could not be reliably determined and then the Friedel pairs were merged and any references to the Flack parameter were removed.

### Crystal data

**Table:**

C_27_H_32_O_2_	*F*(000) = 840
*M**_r_* = 388.53	*D*_x_ = 1.127 Mg m^−^^3^
Monoclinic, *C**c*	Mo *K*α radiation, λ = 0.71073 Å
Hall symbol: C -2yc	Cell parameters from 1555 reflections
*a* = 11.3775 (7) Å	θ = 2.6–32.0°
*b* = 24.6369 (11) Å	µ = 0.07 mm^−^^1^
*c* = 8.8687 (6) Å	*T* = 180 K
β = 112.913 (8)°	Plate, colourless
*V* = 2289.8 (2) Å^3^	0.55 × 0.35 × 0.11 mm
*Z* = 4	

### Data collection

**Table:**

Oxford Diffraction Xcalibur diffractometer	2838 independent reflections
Radiation source: fine-focus sealed tube	1792 reflections with *I* > 2σ(*I*)
graphite	*R*_int_ = 0.048
Detector resolution: 8.2632 pixels mm^-1^	θ_max_ = 28.3°, θ_min_ = 2.6°
ω and φ scans	*h* = −15→13
Absorption correction: multi-scan (*CrysAlis RED*; Oxford Diffraction, 2006)	*k* = −32→32
*T*_min_ = 0.723, *T*_max_ = 1.000	*l* = −8→11
10216 measured reflections	

### Refinement

**Table:**

Refinement on *F*^2^	Secondary atom site location: difference Fourier map
Least-squares matrix: full	Hydrogen site location: inferred from neighbouring sites
*R*[*F*^2^ > 2σ(*F*^2^)] = 0.046	H-atom parameters constrained
*wR*(*F*^2^) = 0.103	*w* = 1/[σ^2^(*F*_o_^2^) + (0.0562*P*)^2^] where *P* = (*F*_o_^2^ + 2*F*_c_^2^)/3
*S* = 0.95	(Δ/σ)_max_ = 0.007
2838 reflections	Δρ_max_ = 0.32 e Å^−^^3^
269 parameters	Δρ_min_ = −0.36 e Å^−^^3^
2 restraints	Extinction correction: *SHELXL97* (Sheldrick, 2008), Fc^*^=kFc[1+0.001xFc^2^λ^3^/sin(2θ)]^-1/4^
Primary atom site location: structure-invariant direct methods	Extinction coefficient: 0.0197 (15)

### Special details

**Table:**

Experimental. Empirical absorption correction using spherical harmonics, implemented in SCALE3 ABSPACK scaling algorithm, CrysAlis RED (Oxford Diffraction, 2006).
Geometry. All e.s.d.'s (except the e.s.d. in the dihedral angle between two l.s. planes) are estimated using the full covariance matrix. The cell e.s.d.'s are taken into account individually in the estimation of e.s.d.'s in distances, angles and torsion angles; correlations between e.s.d.'s in cell parameters are only used when they are defined by crystal symmetry. An approximate (isotropic) treatment of cell e.s.d.'s is used for estimating e.s.d.'s involving l.s. planes.
Refinement. Refinement of *F*^2^ against ALL reflections. The weighted *R*-factor *wR* and goodness of fit *S* are based on *F*^2^, conventional *R*-factors *R* are based on *F*, with *F* set to zero for negative *F*^2^. The threshold expression of *F*^2^ > σ(*F*^2^) is used only for calculating *R*-factors(gt) *etc*. and is not relevant to the choice of reflections for refinement. *R*-factors based on *F*^2^ are statistically about twice as large as those based on *F*, and *R*- factors based on ALL data will be even larger.

### Fractional atomic coordinates and isotropic or equivalent isotropic displacement parameters (Å^2^)

**Table:**

	*x*	*y*	*z*	*U*_iso_*/*U*_eq_	
C1	0.3749 (2)	0.33943 (10)	0.0751 (3)	0.0236 (6)	
H1	0.3948	0.3372	0.1951	0.028*	
C11	0.3659 (3)	0.28073 (10)	0.0172 (4)	0.0282 (6)	
C12	0.2878 (3)	0.26471 (12)	−0.1384 (4)	0.0457 (9)	
H12	0.2378	0.2910	−0.2151	0.055*	
C13	0.2813 (4)	0.21076 (15)	−0.1844 (6)	0.0651 (12)	
H13	0.2276	0.2003	−0.2925	0.078*	
C14	0.3516 (4)	0.17237 (14)	−0.0751 (6)	0.0681 (12)	
H14	0.3459	0.1353	−0.1063	0.082*	
C15	0.4296 (4)	0.18779 (13)	0.0784 (6)	0.0636 (11)	
H15	0.4795	0.1614	0.1544	0.076*	
C16	0.4370 (3)	0.24118 (12)	0.1246 (4)	0.0460 (8)	
H16	0.4920	0.2512	0.2325	0.055*	
C21	0.2487 (3)	0.36963 (10)	0.0021 (3)	0.0256 (6)	
C22	0.1565 (3)	0.36542 (11)	0.0696 (3)	0.0286 (7)	
C23	0.0465 (3)	0.39639 (11)	0.0018 (3)	0.0287 (7)	
H23	−0.0163	0.3942	0.0474	0.034*	
C24	0.0257 (2)	0.43028 (10)	−0.1300 (3)	0.0252 (6)	
C25	0.1125 (3)	0.43357 (10)	−0.2028 (3)	0.0258 (6)	
C26	0.2232 (3)	0.40303 (10)	−0.1329 (3)	0.0248 (6)	
H26	0.2850	0.4051	−0.1802	0.030*	
C31	0.4858 (2)	0.37081 (10)	0.0609 (3)	0.0242 (6)	
C32	0.5345 (2)	0.41658 (10)	0.1601 (3)	0.0247 (6)	
C33	0.6394 (2)	0.44281 (11)	0.1522 (3)	0.0252 (6)	
H33	0.6734	0.4736	0.2197	0.030*	
C34	0.6959 (2)	0.42541 (11)	0.0491 (3)	0.0261 (6)	
C35	0.6492 (2)	0.38059 (11)	−0.0521 (3)	0.0265 (6)	
C36	0.5436 (2)	0.35466 (11)	−0.0424 (3)	0.0244 (6)	
H36	0.5095	0.3241	−0.1108	0.029*	
C221	0.1706 (3)	0.32907 (14)	0.2133 (4)	0.0450 (8)	
H221	0.2512	0.3078	0.2409	0.054*	
C222	0.1828 (5)	0.36190 (19)	0.3634 (5)	0.0802 (14)	
H22A	0.2517	0.3885	0.3867	0.120*	
H22B	0.2022	0.3375	0.4574	0.120*	
H22C	0.1023	0.3809	0.3431	0.120*	
C223	0.0613 (4)	0.28885 (15)	0.1724 (5)	0.0693 (12)	
H22D	0.0777	0.2642	0.2652	0.104*	
H22E	0.0543	0.2678	0.0754	0.104*	
H22F	−0.0185	0.3086	0.1503	0.104*	
C251	0.0870 (3)	0.46852 (13)	−0.3500 (4)	0.0427 (8)	
H25A	0.0060	0.4577	−0.4368	0.064*	
H25B	0.1563	0.4642	−0.3886	0.064*	
H25C	0.0819	0.5066	−0.3210	0.064*	
C321	0.4791 (3)	0.43655 (11)	0.2792 (3)	0.0307 (7)	
H321	0.3979	0.4162	0.2566	0.037*	
C322	0.5693 (3)	0.42314 (13)	0.4530 (4)	0.0475 (9)	
H32A	0.5845	0.3839	0.4631	0.071*	
H32B	0.5312	0.4347	0.5296	0.071*	
H32C	0.6505	0.4421	0.4783	0.071*	
C323	0.4463 (3)	0.49670 (11)	0.2585 (4)	0.0391 (7)	
H32D	0.5242	0.5178	0.2798	0.059*	
H32E	0.4083	0.5075	0.3360	0.059*	
H32F	0.3854	0.5036	0.1465	0.059*	
C351	0.7107 (3)	0.35959 (12)	−0.1625 (4)	0.0357 (7)	
H35A	0.7287	0.3900	−0.2216	0.054*	
H35B	0.6530	0.3339	−0.2412	0.054*	
H35C	0.7907	0.3411	−0.0969	0.054*	
O24	−0.08390 (16)	0.46191 (7)	−0.1939 (2)	0.0328 (5)	
H24	−0.1295	0.4564	−0.1403	0.049*	
O34	0.80030 (17)	0.45253 (7)	0.0405 (2)	0.0346 (5)	
H34	0.8113	0.4819	0.0922	0.052*	

### Atomic displacement parameters (Å^2^)

**Table:**

	*U*^11^	*U*^22^	*U*^33^	*U*^12^	*U*^13^	*U*^23^
C1	0.0212 (14)	0.0255 (14)	0.0257 (15)	0.0017 (11)	0.0109 (12)	0.0019 (11)
C11	0.0210 (13)	0.0262 (14)	0.0406 (17)	−0.0019 (11)	0.0154 (13)	0.0019 (13)
C12	0.0331 (17)	0.0398 (19)	0.054 (2)	0.0041 (14)	0.0065 (15)	−0.0117 (16)
C13	0.0379 (19)	0.058 (2)	0.089 (3)	−0.0044 (18)	0.013 (2)	−0.038 (2)
C14	0.059 (2)	0.0273 (18)	0.132 (4)	−0.0099 (18)	0.052 (3)	−0.021 (2)
C15	0.073 (3)	0.0267 (18)	0.099 (3)	0.0060 (18)	0.041 (3)	0.010 (2)
C16	0.050 (2)	0.0331 (17)	0.057 (2)	0.0036 (15)	0.0232 (17)	0.0079 (16)
C21	0.0251 (14)	0.0213 (13)	0.0322 (16)	−0.0013 (11)	0.0131 (13)	−0.0015 (12)
C22	0.0257 (15)	0.0297 (16)	0.0341 (17)	0.0005 (12)	0.0154 (14)	0.0022 (13)
C23	0.0241 (15)	0.0315 (15)	0.0356 (17)	0.0016 (12)	0.0173 (13)	0.0015 (13)
C24	0.0165 (13)	0.0247 (14)	0.0325 (16)	0.0010 (11)	0.0074 (12)	−0.0016 (13)
C25	0.0255 (14)	0.0239 (14)	0.0291 (15)	−0.0013 (11)	0.0120 (12)	0.0020 (12)
C26	0.0232 (14)	0.0256 (14)	0.0286 (16)	−0.0004 (11)	0.0133 (12)	0.0019 (12)
C31	0.0202 (13)	0.0240 (14)	0.0289 (16)	0.0015 (11)	0.0100 (12)	0.0029 (12)
C32	0.0227 (14)	0.0255 (14)	0.0262 (15)	0.0017 (11)	0.0097 (12)	0.0014 (12)
C33	0.0197 (13)	0.0247 (13)	0.0301 (16)	−0.0014 (11)	0.0083 (12)	−0.0039 (12)
C34	0.0184 (14)	0.0294 (15)	0.0312 (16)	0.0028 (11)	0.0104 (12)	0.0066 (12)
C35	0.0248 (15)	0.0286 (15)	0.0285 (16)	0.0053 (12)	0.0128 (13)	0.0032 (13)
C36	0.0220 (14)	0.0245 (14)	0.0259 (15)	−0.0001 (11)	0.0084 (12)	−0.0012 (12)
C221	0.0374 (19)	0.056 (2)	0.052 (2)	0.0172 (16)	0.0280 (16)	0.0237 (18)
C222	0.097 (3)	0.105 (4)	0.045 (3)	−0.021 (3)	0.034 (2)	0.013 (2)
C223	0.083 (3)	0.049 (2)	0.087 (3)	−0.004 (2)	0.046 (2)	0.026 (2)
C251	0.0353 (17)	0.051 (2)	0.045 (2)	0.0133 (15)	0.0193 (15)	0.0196 (16)
C321	0.0289 (16)	0.0335 (16)	0.0344 (18)	−0.0023 (12)	0.0174 (14)	−0.0064 (13)
C322	0.064 (2)	0.048 (2)	0.0372 (19)	0.0086 (17)	0.0274 (17)	−0.0017 (16)
C323	0.0398 (17)	0.0411 (17)	0.0404 (19)	0.0043 (14)	0.0201 (14)	−0.0082 (15)
C351	0.0346 (17)	0.0420 (18)	0.0348 (18)	−0.0014 (14)	0.0181 (14)	−0.0056 (14)
O24	0.0235 (11)	0.0363 (11)	0.0433 (13)	0.0058 (9)	0.0183 (9)	0.0051 (9)
O34	0.0268 (11)	0.0357 (10)	0.0472 (13)	−0.0063 (9)	0.0209 (10)	−0.0060 (10)

### Geometric parameters (Å, °)

**Table:**

C1—C21	1.520 (4)	C34—C35	1.391 (4)
C1—C11	1.524 (4)	C34—O34	1.390 (3)
C1—C31	1.526 (4)	C35—C36	1.392 (4)
C1—H1	1.0000	C35—C351	1.501 (4)
C11—C12	1.377 (4)	C36—H36	0.9500
C11—C16	1.382 (4)	C221—C222	1.518 (5)
C12—C13	1.384 (4)	C221—C223	1.519 (5)
C12—H12	0.9500	C221—H221	1.0000
C13—C14	1.367 (6)	C222—H22A	0.9800
C13—H13	0.9500	C222—H22B	0.9800
C14—C15	1.359 (6)	C222—H22C	0.9800
C14—H14	0.9500	C223—H22D	0.9800
C15—C16	1.370 (5)	C223—H22E	0.9800
C15—H15	0.9500	C223—H22F	0.9800
C16—H16	0.9500	C251—H25A	0.9800
C21—C26	1.387 (4)	C251—H25B	0.9800
C21—C22	1.399 (4)	C251—H25C	0.9800
C22—C23	1.387 (4)	C321—C322	1.518 (4)
C22—C221	1.514 (4)	C321—C323	1.522 (4)
C23—C24	1.380 (4)	C321—H321	1.0000
C23—H23	0.9500	C322—H32A	0.9800
C24—C25	1.377 (4)	C322—H32B	0.9800
C24—O24	1.391 (3)	C322—H32C	0.9800
C25—C26	1.389 (4)	C323—H32D	0.9800
C25—C251	1.495 (4)	C323—H32E	0.9800
C26—H26	0.9500	C323—H32F	0.9800
C31—C36	1.377 (4)	C351—H35A	0.9800
C31—C32	1.404 (3)	C351—H35B	0.9800
C32—C33	1.383 (4)	C351—H35C	0.9800
C32—C321	1.508 (4)	O24—H24	0.8400
C33—C34	1.375 (4)	O34—H34	0.8400
C33—H33	0.9500		
			
C21—C1—C11	113.1 (2)	C34—C35—C351	122.4 (2)
C21—C1—C31	113.04 (19)	C36—C35—C351	121.0 (2)
C11—C1—C31	113.8 (2)	C31—C36—C35	123.7 (2)
C21—C1—H1	105.3	C31—C36—H36	118.1
C11—C1—H1	105.3	C35—C36—H36	118.1
C31—C1—H1	105.3	C22—C221—C222	111.5 (3)
C12—C11—C16	117.7 (3)	C22—C221—C223	112.1 (3)
C12—C11—C1	122.8 (3)	C222—C221—C223	110.1 (3)
C16—C11—C1	119.5 (3)	C22—C221—H221	107.7
C11—C12—C13	120.7 (3)	C222—C221—H221	107.7
C11—C12—H12	119.7	C223—C221—H221	107.7
C13—C12—H12	119.7	C221—C222—H22A	109.5
C14—C13—C12	120.4 (4)	C221—C222—H22B	109.5
C14—C13—H13	119.8	H22A—C222—H22B	109.5
C12—C13—H13	119.8	C221—C222—H22C	109.5
C15—C14—C13	119.4 (3)	H22A—C222—H22C	109.5
C15—C14—H14	120.3	H22B—C222—H22C	109.5
C13—C14—H14	120.3	C221—C223—H22D	109.5
C14—C15—C16	120.5 (4)	C221—C223—H22E	109.5
C14—C15—H15	119.7	H22D—C223—H22E	109.5
C16—C15—H15	119.7	C221—C223—H22F	109.5
C15—C16—C11	121.3 (4)	H22D—C223—H22F	109.5
C15—C16—H16	119.4	H22E—C223—H22F	109.5
C11—C16—H16	119.4	C25—C251—H25A	109.5
C26—C21—C22	118.2 (2)	C25—C251—H25B	109.5
C26—C21—C1	120.2 (2)	H25A—C251—H25B	109.5
C22—C21—C1	121.5 (2)	C25—C251—H25C	109.5
C23—C22—C21	118.3 (2)	H25A—C251—H25C	109.5
C23—C22—C221	118.2 (3)	H25B—C251—H25C	109.5
C21—C22—C221	123.5 (2)	C32—C321—C322	109.7 (2)
C24—C23—C22	121.9 (3)	C32—C321—C323	112.5 (2)
C24—C23—H23	119.1	C322—C321—C323	111.8 (2)
C22—C23—H23	119.1	C32—C321—H321	107.5
C25—C24—C23	121.0 (2)	C322—C321—H321	107.5
C25—C24—O24	118.0 (2)	C323—C321—H321	107.5
C23—C24—O24	121.0 (2)	C321—C322—H32A	109.5
C24—C25—C26	116.7 (2)	C321—C322—H32B	109.5
C24—C25—C251	120.8 (2)	H32A—C322—H32B	109.5
C26—C25—C251	122.4 (3)	C321—C322—H32C	109.5
C25—C26—C21	123.7 (3)	H32A—C322—H32C	109.5
C25—C26—H26	118.1	H32B—C322—H32C	109.5
C21—C26—H26	118.1	C321—C323—H32D	109.5
C36—C31—C32	118.4 (2)	C321—C323—H32E	109.5
C36—C31—C1	122.1 (2)	H32D—C323—H32E	109.5
C32—C31—C1	119.5 (2)	C321—C323—H32F	109.5
C33—C32—C31	118.7 (2)	H32D—C323—H32F	109.5
C33—C32—C321	119.2 (2)	H32E—C323—H32F	109.5
C31—C32—C321	122.1 (2)	C35—C351—H35A	109.5
C34—C33—C32	121.6 (2)	C35—C351—H35B	109.5
C34—C33—H33	119.2	H35A—C351—H35B	109.5
C32—C33—H33	119.2	C35—C351—H35C	109.5
C33—C34—C35	121.1 (2)	H35A—C351—H35C	109.5
C33—C34—O34	121.1 (2)	H35B—C351—H35C	109.5
C35—C34—O34	117.8 (2)	C24—O24—H24	109.5
C34—C35—C36	116.5 (2)	C34—O34—H34	109.5

### Hydrogen-bond geometry (Å, °)

**Table:**

*Cg*2 and *Cg*3 are the centroids of the C21–C26 and C31–C36 rings, respectively.

**Table:**

*D*—H···*A*	*D*—H	H···*A*	*D*···*A*	*D*—H···*A*
O24—H24···O34^i^	0.84	2.05	2.871 (3)	164
O34—H34···O24^ii^	0.84	2.27	3.051 (3)	154
C23—H23···O34^i^	0.95	2.51	3.259 (3)	135
C13—H13···Cg3^iii^	0.95	2.92	3.658 (4)	135
C15—H15···Cg2^iv^	0.95	2.86	3.790 (5)	167

Symmetry codes: (i) *x*−1, *y*, *z*; (ii) *x*+1, −*y*+1, *z*+1/2; (iii) *x*−1/2, −*y*+1/2, *z*−1/2; (iv) *x*+1/2, −*y*+1/2, *z*+1/2.
